# Non-conscious processing of fear faces: a function of the implicit self-concept of anxiety

**DOI:** 10.1186/s12868-023-00781-9

**Published:** 2023-02-05

**Authors:** Vivien Günther, Jonas Pecher, Carolin Webelhorst, Charlott Maria Bodenschatz, Simone Mucha, Anette Kersting, Karl-Titus Hoffmann, Boris Egloff, Donald Lobsien, Thomas Suslow

**Affiliations:** 1 Department of Psychosomatic Medicine and Psychotherapy, University of Leipzig Medical Center, 04103 Leipzig, Germany; 2 Department of Neuroradiology, University of Leipzig Medical Center, 04103 Leipzig, Germany; 3 Department of Psychology, Johannes Gutenberg University of Mainz, 55122 Mainz, Germany

**Keywords:** MRI, fMRI, Implicit anxiety, Trait anxiety, Subliminal, Non-conscious perception

## Abstract

**Background:**

Trait anxiety refers to a stable tendency to experience fears and worries across many situations. High trait anxiety is a vulnerability factor for the development of psychopathologies. Self-reported trait anxiety appears to be associated with an automatic processing advantage for threat-related information. Self-report measures assess aspects of the explicit self-concept of anxiety. Indirect measures can tap into the implicit self-concept of anxiety.

**Methods:**

We examined automatic brain responsiveness to non-conscious threat as a function of trait anxiety using functional magnetic resonance imaging. Besides a self-report instrument, we administered the Implicit Association Test (IAT) to assess anxiety. We used a gender-decision paradigm presenting brief (17 ms) and backward-masked facial expressions depicting disgust and fear.

**Results:**

Explicit trait anxiety was not associated with brain responsiveness to non-conscious threat. However, a relation of the implicit self-concept of anxiety with masked fear processing in the thalamus, precentral gyrus, and lateral prefrontal cortex was observed.

**Conclusions:**

We provide evidence that a measure of the implicit self-concept of anxiety is a valuable predictor of automatic neural responses to threat in cortical and subcortical areas. Hence, implicit anxiety measures could be a useful addition to explicit instruments. Our data support the notion that the thalamus may constitute an important neural substrate in biased non-conscious processing in anxiety.

**Supplementary Information:**

The online version contains supplementary material available at 10.1186/s12868-023-00781-9.

## Background

Anxiety is a basic emotional state that is characterized by high arousal, vigilance, and the subjective experience of tension elicited by distant and less predictable threats [1]. Spielberger et al. [2] have proposed a distinction between state and trait aspects of anxiety. State anxiety describes an emotional state that is characterized by feelings of tension and apprehension, and heightened autonomic activity in situations that are perceived as threatening. This condition can alternate and vary in intensity. In contrast, trait anxiety is defined as a disposition to experience anxiety and respond fearfully to various unspecific threatening situations [2]. Trait anxiety is considered a relatively stable personality characteristic. Although trait and state anxiety are typically related [2], both constructs were shown to differentially influence cognitive functions and brain responsiveness (e.g., [3–5]). Trait-anxious individuals appear to be highly susceptible to stress and have a higher risk for the development of stress-induced psychopathologies [6]. Trait anxiety has been identified as a risk factor that predisposes anxiety disorders and depression [7–9]. High trait anxious individuals exhibit attentional preferences for threat stimuli [10] and demonstrate a facilitated threat detection [5], consistent with a mood-congruent bias in emotion perception (e.g., [11]). It has been suggested that non-conscious neural processes may underlie the observed information processing biases in high trait anxiety [12, 13]. In the anxiety model of Beck and Clark [14], it is proposed that the initial stage of biased information processing operates at an automatic level, which involves the involuntary, rapid, and non-conscious recognition of stimuli. Also, Mathews and Mackintosh [15] have suggested an automatic and nonconscious evaluation system for the threat value of a stimulus, which is more sensitive and negatively biased during anxious arousal. Thus, the frequent experience of anxious states in trait anxiety may be assumed to prime an individual’s perception of potential harm. In line with the assumptions of Beck and Clark [14] and Mathews and Mackintosh [15], high trait anxiety was shown to be associated with an exaggerated activation in the amygdala in response to non-consciously processed fearful faces during a color-decision task [12, 13]. The amygdala is involved in threat detection and evaluation [16, 17], and in the recruitment of attentional resources for the processing of salient stimuli [18, 19]. In high trait anxiety, the amygdala is considered as part of a hypersensitive appraisal circuit [20], that exerts an influence on biased threat processing [21, 22]. In other anxiety models, an imbalance between increased stimulus-driven threat detection in the amygdala and impaired attentional control processes in the prefrontal cortex has been suggested as a mechanism that underlies biased threat processing ([4, 21], see [23] for an overview). By extending these models, Sussmann et al. [24] and Grupe and Nitschke [22] highlighted the importance of top-down processes, such as exaggerated anticipations regarding the occurrence of negative events, which may drive the prioritized threat perception in anxiety. An extensive overview for cognitive models in anxiety is provided by Mogg and Bradley [25].

Only few studies investigated brain responses during automatic (i.e., non-conscious, fast, efficient, or unintentional, see [26]) processing of threat faces as a function of non-clinical trait anxiety. The results of some studies are partly consistent with findings of Etkin et al. [12] and Günther et al. [13] of altered automatic amygdala activation. In highly anxious individuals, an amygdalar hyper-responsiveness to unattended [27], and very briefly displayed [28] fearful faces has been found.

In a gender-decision task with clearly visible angry and fearful faces [29] and in an implicit processing task with threat pictures [30] no relation among trait anxiety and automatic amygdala activation was reported. However, increased activations in the precuneus and medial prefrontal cortex were revealed in high trait anxious individuals [29]. In a perceptual load task with fearful faces as distractor stimuli, Bishop et al. [4] demonstrated an impact of high trait anxiety on decreased neural responses in the anterior cingulate and lateral prefrontal cortex. This hypo-activation has been discussed as a potential indicator of weakened attentional control. In a color-identification task, increased lateral prefrontal and temporal activity in response to subliminal threat [12], but not to clearly visible threat (see supplement of [13]), has been observed in highly anxious individuals. During passive viewing and thus, the unintentional and goal-irrelevant perception of clearly visible threat-related scene pictures, Brinkmann et al. [31] found no association between brain activity and trait anxiety. Thus, evidence for an overall automatic processing advantage for threat-related stimuli in the amygdala or cortical brain areas is rather inconsistent. Although earlier findings are heterogeneous, prior research on non-clinical anxiety suggests that alterations in the responsivity of the amygdala, as well as in areas involved in perception of emotional stimuli, might underlie trait anxiety.

Notably, in all cited face processing studies [4, 12, 13, 27–29], fearful or angry faces were used as threat-related stimuli. The neglect of facial expressions of disgust as threatening stimuli is surprising, given that disgust, as well as anger and contempt, has been considered a main affective component of hostility [32]. The facial expression of disgust signals rejection and revulsion [33]. Interestingly, Schienle et al. [34] demonstrated an effect of trait anxiety on neural disgust responses in the amygdala and insula by using a passive viewing task with disgusting pictures.

The predominant measure of trait anxiety in previous imaging studies is the State-Trait-Anxiety-Inventory (STAI; [2]). The STAI is a self-report measure that assesses conscious or propositional representations of one’s own anxious experiences, i.e., the explicit self-concept of anxiety. Explicit measures of personality characteristics rely on reflective reasoning processes and presuppose introspective access to self-related knowledge [35]. However, conscious awareness of one’s own emotional reactions can be restricted [36]. Therefore, indirect assessment methods for affect and personality have been introduced [37, 38]. A well-known and validated indirect measure of personality characteristics is the Implicit Association Test (IAT; [39]). In general, the IAT assesses the strength of automatic mental associations between target concepts (e.g., the self) and attribute dimensions (e.g., “afraid” or “nervous”) by comparing response times in two word classification tasks. Egloff and Schmukle [37] adapted the original IAT to provide an implicit measure of anxiety. It has been argued that the recurrent performance of (anxious) behaviors and reactions that were driven by automatic tendencies become manifest in the implicit self-concept of anxiety [40], as measured by the IAT. Behaviors that are triggered by more deliberate processes should result in the development of the explicit self-concept of anxiety, as measured by self-report. It has been shown that the IAT can explain variance in behavioral anxiety indicators above and beyond explicit measures [37, 40–42]. Implicit and explicit self-concepts of anxiety appear to share only a small amount of common variance [37]. Therefore, the additional administration of indirect measures of anxiety can be promising to increase the prediction of spontaneous anxious behaviors and neurobiological anxiety responses.

To our knowledge, only two previous studies investigated brain responsiveness to threat faces as a function of implicit anxiety. By using the IAT, Suslow et al. [43] found neither explicit nor implicit anxiety to be predictive of brain activity during controlled facial affect processing in an emotion recognition task. Günther et al. [13] reported a relationship among implicit anxiety and automatic brain response in the amygdala and fusiform gyrus. Here, implicit anxiety was assessed by the Implicit Positive and Negative Affect Test (IPANAT, [38]), a questionnaire where anxiety was inferred from the extent to which artificial nonsense words are judged to express anxiety.

In the present study, we used functional magnetic resonance imaging in healthy individuals. For the first time we investigated automatic brain responses to facial expressions of fear and disgust as a function of the implicit self-concept of anxiety. To this aim, we used a gender-decision task with briefly presented, backward-masked emotional faces. In contrast to Suslow et al. [43], we chose a paradigm where emotional faces were processed below the threshold of conscious awareness. It was hypothesized that the implicit self-concept of anxiety is positively related to automatic brain responsiveness to threat (i.e., fear and disgust) stimuli, particularly in the amygdala, independent of directly measured anxiety.

## Experimental procedures

### Participants and psychometric measures

Forty one healthy volunteers took part in our study. All participants were native German speakers and had normal or corrected-to-normal visual acuity. Participants were recruited via online advertisement in social networks and public notices that were posted in canteens, libraries and student halls of residence. A history of psychiatric or neurological diseases, head trauma involving loss of consciousness, left-handedness, and contraindications for magnetic resonance imaging (MRI) were exclusion criterions for study participation. Diagnoses of past or current Axis I disorders were determined with the Structured Clinical Interview for DSM-IV Axis I disorders (SCID-I; [44]). Trait anxiety was measured with the trait version of the State-Trait Anxiety Inventory (STAI; [45]).

The aim of this study was to investigate non-conscious processing of threatening faces as a function of the implicit self-concept of anxiety. Therefore, individuals with an objective awareness for masked and briefly presented threat faces were excluded from data analyses (“Objective visibility check for masked threat faces” section). This procedure resulted in a final sample of *N* = 37 subjects (19 women), with a mean age of 23.89 years (*SD* = 3.83) and a mean school education of 12.14 years (*SD* = 0.35, range: 12–13). Questionnaire characteristics of the final sample are presented in Table 1.

**Table 1:** Descriptive statistics and correlations between psychometric measures

	*M* (*SD*)	Anxiety	Performance	
		IAT	RT-effect disgust	RT-effect fear
STAI-T	35.30 (6.95)	− 0.02	− 0.06	0.12
IAT anxiety	− 0.46 (0.31)	–	0.25	0.09
RT-effect disgust	0.77 (34.94)	–	–	0.58*
RT-effect fear	8.90 (30.94)	–	–	–

*p < 0.01 (two-tailed)

Participants received financial compensation after completion of all tasks.

### Anxiety-IAT

To assess implicit anxiety, an IAT following the procedure of Egloff and Schmukle [37] was administered. The anxiety-IAT is a well-validated indirect measure with a satisfactory internal consistency [37, 46]. Participants were instructed to make a series of category judgments as accurate and quickly as possible. On each trial, single-word stimuli were presented in the center of the screen, whereas category labels were presented on the left and right. Participants were given the task to assign the word stimulus to the correspondent category via two response keys on a keyboard (“Q” and “P” button). The experiment included stimuli for the target categories “self” (I, me, my, own, self) vs. “others” (they, them, you, your, others) and for the attribute categories “anxiety” (anxious, afraid, nervous, fearful, uncertain) vs. “calmness” (balanced, at ease, calm, restful, relaxed). The IAT comprised five blocks including two critical and three practice blocks. In practice Block 1 (20 trials), participants differentiated words from the “self” category from words of the “others” category. In practice Block 2 (20 trials), participants had to sort words into anxiety and calmness categories. Block 3 and 5 are the critical blocks (60 trials each) in which participants were required to sort items of two combined categories, each including the attribute and the target concept that were assigned to the same key. In Block 3 (self + anxiety block) the target concept category “self” and the attribute concept category “anxiety” were assigned to the left key, whereas “others” and “calmness” were assigned to the right key. In Block 5 (self + calmness block) the assignment of the attribute concept categories “anxiety” vs. “calmness” was inversed, so “self” and “calmness” were combined on the left key, and “others” and “anxiety” were combined for the right key. In practice block 4 (20 trials), the switched key assignment for the anxiety and calmness category was trained. An illustration of the IAT can be seen in Additional file 1: Fig. S1. Inquisit 3 [47] was used to control stimulus presentation and to record task performance. Following a standard computation method of previous studies [43, 46], the improved D_1_ scoring algorithm was used to compute IAT scores (see [48]). Here, trials with latencies greater than 10,000 ms were deleted and error trials were retained in the analysis. Within the experiment, participants received an error feedback and were required to correct false responses (built-in error penalty). For each subject, mean latency for trials of the critical Block 3 (self + anxiety) was subtracted from the mean latency for trials of the critical Block 5 (self + calmness). The IAT effect was calculated by dividing the difference score by the individual-respondents standard deviation of all latencies in both sorting conditions. Higher (more positive) IAT effects indicate a more anxious implicit self-concept. Reliability was calculated by correlating IAT effects for odd and even trials and by applying a Spearman-Brown correction. The reliability coefficient was 0.73 in the present sample.

### fMRI experiment: gender-decision task

Stimuli of the masked face processing task consisted of photographs of 72 actors (36 women). They depicted either fearful, disgusted, happy or neutral facial expressions, chosen from the FACES database [49]. Participants were instructed to respond to the perceived gender of presented faces as accurately and quickly as possible. Participants held fiber optic response pads in both hands with two buttons each, and responses were given via the left and right index finger.

**Fig. 1:**
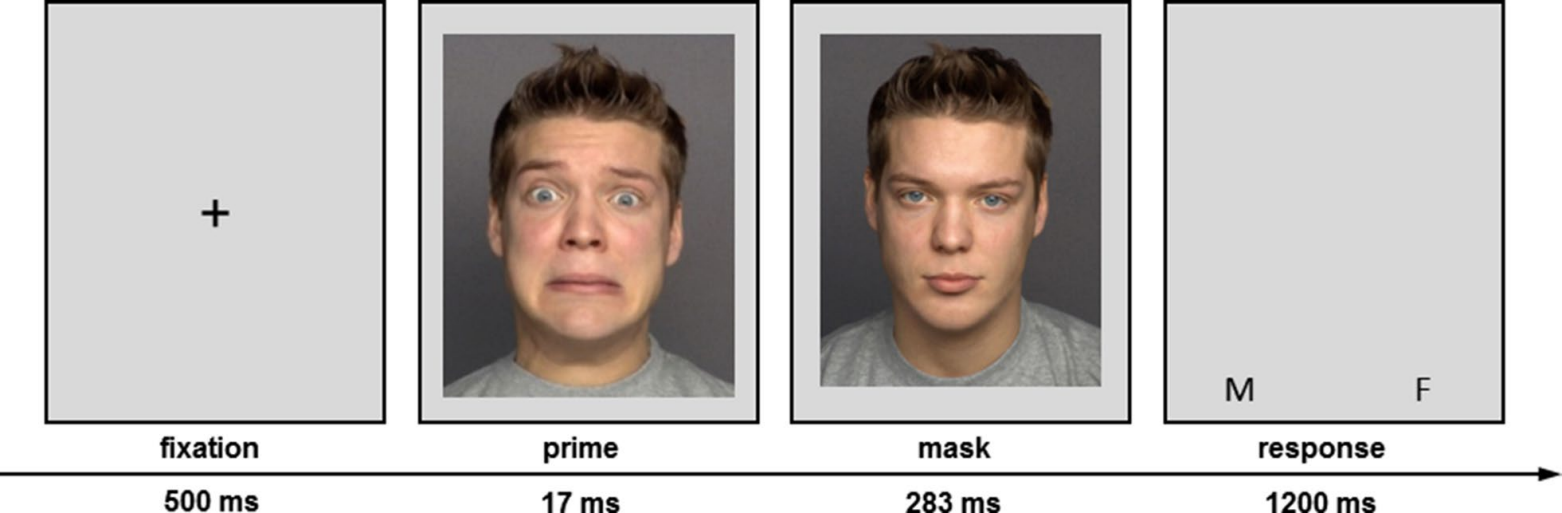
FMRI paradigm. Depicted is the sequence of events within a trial of the gender-decision task. Prime stimuli were disgusted, fearful, or neutral faces masked by a neutral face of the same actor. The two images shown stem from the Lifespan Database of Adult Emotional Facial Stimuli FACES [43] and are publicly available

Each trial lasted for 2 s, see Fig. 1. It started with a fixation cross shown for 500 ms, followed by a brief (17 ms) prime face, which was immediately masked by a neutral target face of the same actor for 283 ms. Subsequently, a gray screen depicting the assignment of the gender responses to the buttons was presented for 1.2 s. Participants had to give their response in this time frame.

Prime faces displayed fearful, disgusted, happy, and neutral expressions. Identity of prime and mask in the neutral face condition was avoided by using vertically mirrored versions of the neutral mask face as prime face. The task comprised 24 blocks (6 per condition) of 6 trials.

Within a block, the emotion was constant and trials were randomized with respect to gender, but presentation frequencies of actors were balanced across the experiment. We chose two fixed counterbalanced sequences for all trials to avoid stimulus order effects. Each block had a duration of 12 s and was followed by a 12 s blank screen. The experiment consisted of 144 trials and lasted for approximately 10 min. Presentation^®^ software (Version 16.3, Neurobehavioral Systems, Inc., Berkeley, CA, www.neurobs.com) was used to control stimulus presentation and to record responses.

Of note, the happy condition was only included to ensure a more balanced design regarding a positive and negative stimulation throughout the experiment. Since the processing of happy faces as a function of anxiety was not of interest in this study, results for the happy condition are not reported.

### Objective visibility check for masked threat faces

Participants’ objective awareness of briefly presented masked fearful and disgust faces was examined. Therefore, after the fMRI scanning a forced-choice detection task was administered. The presentation conditions were identical to the conditions during scanning, with the exception that participants additionally had to label the presented emotion. Subjects were informed about the presence of briefly presented emotional or neutral faces. They were instructed to indicate for each trial the gender of the face and afterwards, which emotion was presented. Following the procedure of earlier studies (e.g., [13]) performance above chance for the emotion recognition was determined according to the one-tailed (*p* < 0.05) binomial model for each emotional condition. A more detailed description of the procedure can be found in the Additional file 1. According to this procedure, subjects with accuracy higher than 33.3% were considered as objectively aware of the respective emotion, since the recognition performance was significantly above chance level. Four participants exceeded the threshold for recognizing masked fearful faces. They were excluded from further analyses to ensure non-conscious processing of threat faces. No participant exceeded the threshold for the recognition of disgust faces. Of note, detection accuracy for disgust and fearful faces was not related to explicit (disgust: *r*(39) = 0.01, *p* = 0.97, fear: *r*(39) = 0.03, *p* = 0.84) or implicit self-concept of anxiety (disgust: *r*(39) = 0.07, *p* = 0.68, fear: *r*(39) = 0.11, *p* = 0.50) in the initial sample. Thus, the exclusion of aware subjects did not systematically concern particularly high anxious individuals.

### MRI acquisition and preprocessing

Structural and functional MR images were acquired using a 3T scanner (Magnetom Trio, Siemens, Erlangen, Germany) with a 20-channel coil. Structural images were obtained with a T1-weighted 3D MP-RAGE [50], with the following imaging parameters TI 900 ms, TR 1900 ms, TE 2.65 ms, flip angle 9°, spatial resolution of 0.8 × 0.8 × 1 mm^3^, two averages. Blood oxygen level dependent (BOLD) contrast sensitive images were collected using T2*-weighted echo-planar imaging (EPI) sequence [matrix 64^2^; resolution 3.5 × 3.5 × 3.5 mm^3^; TR 2.54 s; TE 30 ms; flip angle 90°; interleaved acquisition of 40 slices along the AC-PC plane; 237 images]. SPM8 (http://www.fil.ion.ucl.ac.uk/spm/) was used to preprocess and analyze MRI data. The first four functional volumes were discarded to allow longitudinal magnetization to reach equilibrium. Preprocessing included slice time-correction, motion-correction, and co-registration. Anatomical images were segmented, including normalization to the Montreal Neurological Institute (MNI) template. The normalization parameters were then applied to the functional EPI series (resulting in a re-sampled voxel size of 3 × 3 × 3 mm^3^). A temporal high-pass filter (128 s) was applied. Functional data were smoothed (Gaussian kernel size = 6 mm).

### Data analyses

For reaction times (RT) in the gender-decision task, only trials with correct responses were analyzed. Mean accuracy was 97% (*SD* = 0.03%) and error rates were not correlated with the STAI or IAT (*r*(35) = 0.09, *p* = 0.62 and *r*(35) = −0.03, *p* = 0.84, respectively). RT difference scores (RT-effects) were calculated by subtracting mean RTs for neutral from disgust and fearful trials, each separately. According to Etkin et al. [12] the RT-effects can be considered as measures of attention allocation, with lower scores indicating enhanced performance (faster reactions) for threat faces as compared to neutral faces.

Functional MRI data were analyzed by modeling the onset and duration of 12 s for each block. Regressors were convolved with a hemodynamic response function for the four conditions (disgust, fear, happy, neutral). First level *t*-contrasts were calculated by contrasting the disgust, fear, and happy condition to the neutral one, each respectively. These contrasts allow clear conclusions whether activation can be uniquely attributed to the emotional content of facial expressions.

For the second level analyses, parametric one-sample *t*-tests were performed in SPM to determine main effects of masked threat expressions (vs. neutral ones). Additionally, contrast images were entered into parametric regression models in SPM with the individual STAI and IAT scores as regressors of interest. One regression model was calculated per anxiety measure. Our experiment was based on minimal stimulation with prime faces being presented for only one sixtieth of a second (17 ms). This subtle stimulus presentation was not expected to elicit large activation differences with varying explicit and implicit anxiety. Thus, exploratory whole-brain analyses in SPM were conducted with a voxel-wise threshold at *p* < 0.005 (uncorrected) and an additional cluster-level threshold of *p* < 0.05, family-wise-error (FWE) corrected. ROI analyses in SPM were carried out for the bilateral amygdala. To create an anatomically defined mask, the WFU Pickatlas [51] was used (according to [52]). For the ROI analysis in SPM, the statistical threshold was set to *p* = 0.05, FWE-corrected. Subsequently, for each participant the averaged signal (contrast estimates) across a significant cluster, that was revealed in SPM whole-brain analyses, or across significant voxels of the ROI analyses, were extracted by using the MarsBaR toolbox, see [53]. These extracted mean values were used to calculate further analyses in SPSS25. We conducted these additional two-stage hierarchical regression analyses in SPSS to rule out whether implicit anxiety (IAT) can explain an incremental proportion of variance in brain responsiveness, in addition to explicit anxiety (STAI). In a first step, STAI scores and gender were entered as predictors in the model to regress out a possible influence. In a second step, IAT scores were included as predictor of interest. The residuals of the calculated regression models were normally distributed (K-S-Lilliefors test, all *p*s > 0.05). In our Additional file 1, parametric (Pearson *r*) and non-parametric (Spearman *r*_s_) correlations between the IAT and the extracted averaged signals from significant clusters were calculated to compare both coefficients. We could demonstrate that parametric and non-parametric analyses revealed results that did not significantly differ from each other.

## Results

### Reaction times

The STAI and IAT were not significantly correlated with RT-effects, see Table 1.

### Neural response

#### Main effects of masked disgust and fearful faces

Masked disgust faces (vs. neutral faces) produced marginally significant brain activation in the left cuneus (BA 18 and BA19): peak voxel xyz: −3 −82 28, *T* = 3.96, *p* < 0.001, cluster size: 150, cluster *p*_FWE_ = 0.08).

Masked fearful faces (compared to neutral faces) significantly evoked activation in a large cluster including the left supramarginal gyrus, left precentral gyrus (BA6), left supplemental motor area, left postcentral gyrus, and left middle and superior frontal gyrus (peak voxel xyz: −30 2 64, *T* = 4.97, *p* < 0.001, cluster size: 858, cluster *p*_FWE_ < 0.001). Additionally, significant activations were found in a large cluster including the bilateral thalamus (pulvinar), right parahippocampal gyrus, left middle temporal gyrus, and bilateral posterior cingulate gyrus (peak voxel xyz: 9 −31 7, *T* = 4.58, *p* < 0.001, cluster size: 896, cluster *p*_FWE_ < 0.001). For interested readers, findings for a less stringent significance threshold on the cluster-level (*p* < 0.05 *un*corrected, with *k* > 82 voxels) can be seen in Additional file 1: Table S1. Of note, the amygdala was not included in the activated clusters.

#### Relation among brain activity to masked threat faces and explicit anxiety

SPM whole-brain and ROI-based regression analyses with the STAI yielded no significant clusters above the statistical threshold for masked disgust or fearful faces (vs. neutral faces). Non-significant findings that were revealed at an extremely lenient threshold of *p* < 0.05 (uncorrected) and k > 10 voxel are provided in Additional file 1: Table S3.

#### Relation among brain activity to masked threat faces and implicit anxiety

SPM exploratory whole-brain regression analyses and ROI-analyses with the IAT did not reveal associations with brain reactivity to masked disgust faces (vs. neutral faces). Non-significant findings that were revealed at an extremely lenient threshold of *p* < 0.05 (uncorrected) and k > 10 voxel are provided in Additional file 1: Table S3.

**Fig. 2:**
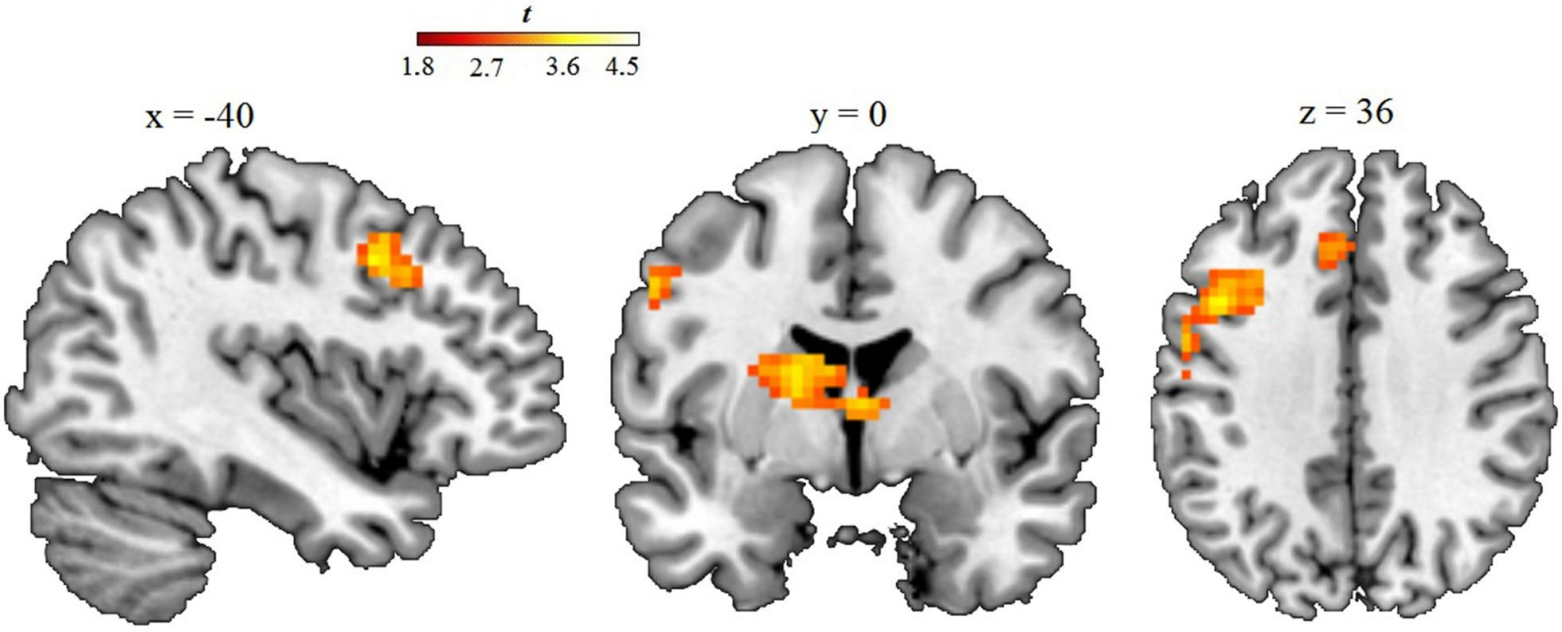
Results from whole-brain regression analyses with implicit anxiety (IAT) predicting brain responsiveness to masked fearful faces. Sagittal, coronal, and axial images in neurological orientation showing the relationship among implicit anxiety and increased activity in response to non-conscious fearful faces in the thalamus and left frontal gyrus. The voxel-wise threshold was set to *p* = 0.005 (uncorrected) with a cluster-level threshold of *p* < 0.05, FWE-corrected. Color bar: *t*-values

In SPM exploratory whole-brain regression analyses for masked fearful (vs. neutral) faces, the IAT positively correlated with activity in the bilateral thalamus (peak voxel xyz: −6 −13 −1, *T* = 4.66, *p* < 0.001, cluster size: 263, cluster *p*_FWE_ = 0.014). Moreover, the IAT was significantly and positively associated with brain activity to masked fear in the left precentral gyrus, extending to the middle and superior frontal gyrus (BA6,8,9), (peak voxel xyz: −42 8 40, *T* = 4.18, *p* < 0.001, cluster size: 299, cluster *p*_FWE_ = 0.01), see Fig. 2. Mean activations in the clusters located in the bilateral thalamus and in the frontal gyrus were extracted for additional hierarchical regression analyses in SPSS. In the first step, variance in thalamus activity was not significantly explained by gender (*β* = −0.14; *p* = 0.43) or the STAI (*β* = 0.06; *p* = 0.73), (*R*^*2*^ = 0.02; *F*(2,34) = 0.38, *p* = 0.69). Also for the frontal gyrus, gender and STAI did not significantly contribute to the regression model in the first step (*β* = 0.03; *p* = 0.88 and *β* = 0.18; *p* = 0.30, respectively), (*R*^*2*^ = 0.03; *F*(2,34) = 0.58, *p* = 0.57). Entering implicit anxiety (IAT) in the second step for the thalamus and frontal gyrus did significantly increase the predictive values of both models (*ΔR*^*2*^ = 0.31, *p* < 0.001; *F*(3,33) = 5.49, *p* = 0.004 and *ΔR*^*2*^ = 0.34, *p* < 0.001; *F*(3,33) = 6.55, *p* = 0.001, respectively). Hence, implicit anxiety remained a significant predictor of brain activation even after accounting for the effect of gender and explicit anxiety.

SPM ROI-based analyses revealed no significant association between IAT and amygdala activation. Results of the whole-brain regression analyses in SPM for a less stringent significance threshold on the cluster-level (*p* < 0.05 *un*corrected, with *k* > 82 voxels) can be seen in Additional file 1: Table S2.

## Discussion

We investigated automatic brain responsiveness to masked threat faces as a function of the implicit self-concept of anxiety. For the first time, the IAT and STAI were administered to a sample of young and healthy adults to determine associations with brain responsiveness to very briefly presented (17 ms) masked disgust and fearful faces. We aimed to shed light on the implicit self-concept of anxiety as a potential predictor of automatic brain responsiveness to subliminal threat stimuli, after controlling for an influence of explicit anxiety. Implicit and explicit anxiety were not significantly correlated, indicating that they refer to rather distinct aspects of anxiety (see also [37]). Explicit trait anxiety was not related to brain responsivity to subliminally presented disgust or fearful faces. However, individuals with a more anxious implicit self-concept showed increased activations to fearful faces in the thalamus and a cluster including the precentral gyrus and middle and superior frontal gyrus, but not in the amygdala. The relationships among implicit anxiety and brain activation remained significant when accounting for a potential influence of explicit trait anxiety.

In contrast to Schienle et al. [34], who presented disgusting pictures, we did not reveal an effect of explicit or implicit self-concept of anxiety on neural responses to masked facial expressions of disgust. Disgust has been discussed as an affective component of hostility [32] that could signal negative evaluation or interpersonal rejection [54]. It is conceivable that disgust faces are highly threatening in particular for socially anxious individuals [55, 56], but have a lower relevance for individuals with more general anxious tendencies.

Fearful faces are considered as biologically salient signals of potential threats in the environment [57] and were shown to elicit faster responses in the amygdala than neutral and happy faces or unpleasant scenes [58]. Given their adaptive survival value, the processing of fearful faces appears to have a privileged status in the brain [59]. In our study, masked fearful faces, that were presented briefly and were not consciously perceived, induced higher activity compared to neutral faces in a wide brain network including the thalamus, middle temporal and posterior cingulate gyrus, the supramarginal gyrus, pre- and postcentral gyrus, and middle and superior frontal gyrus.

The temporal and frontal gyri were shown to be engaged in emotion and face processing [60, 61]. Automatic reactivity in these brain areas to masked faces with ambiguous or fearful expressions have been previously observed [59, 62–64]. In our study, increased thalamic, precentral, and prefrontal activation was also modulated by the implicit anxiety, with anxious individuals showing heightened reactivity in these brain areas. Thus, individuals who exhibit stronger associations of the self with anxiety-related characteristics, such as nervousness, fearfulness, and uncertainty demonstrated exaggerated brain responsiveness to subliminal fearful faces. The thalamus is a subcortical brain structure that is substantially interconnected with the amygdala and the prefrontal and tempo-parietal cortex [65, 66] and has been considered as an important node in the neural anxiety network [67]. It operates at an early stage in the visual encoding of salient stimuli [68]. The thalamus directly conveys coarse threat-related information to the amygdala [69, 70]. This pathway enables rapid processing of and automatic adaptive reactions to potential dangers, which need not to be accessible to conscious awareness (see also [71] for an overview). Therefore, the thalamus assists the initial detection of threats. Liddell et al. [63] highlighted the thalamus and amygdala as part of an automatic alerting system in response to danger signals, which does not rely on conscious appraisal of threat stimuli. In line with this notion, Kanat et al. [72] demonstrated increased reactivity in the thalamus and amygdala to non-consciously processed fearful eyes. The thalamus contributes to the selection of relevant information from the environment and mediates awareness of visual information [73, 74]. Carretié et al. [75] have suggested a crucial role of thalamic nuclei for a fast evaluation of salient stimuli. Our results provide further evidence for the involvement of the thalamus in the non-conscious perception of threat (see [68] for an overview). Activity in the thalamus appears to vary as a function of the implicit self-concept of anxiety. Given its role in processing emotional stimuli and in mediating attentional processes, heightened responsiveness in the thalamus in individuals with a more anxious implicit self-concept may indicate an increased sensitivity for threat and a higher capacity for the maintenance of attention to potential dangers. This is in line with findings of Geng et al. [76], who observed in high trait anxiety heightened activity in the thalamus during the anticipation of uncertain threat.

It is assumed that the implicit self-concept of personality characteristics, such as trait anxiety or neuroticism, becomes manifest through the recurrent performance of non-controlled and spontaneous behavior that is driven by impulsive processes, such as nervous gestures or physiological responses [40]. Therefore, the IAT measures anxious action tendencies that are not necessarily accessible to consciousness. In line with our findings on automatic brain responsiveness, the IAT was shown to be predictive of actual anxious behaviors that are not entirely subject to conscious control [37, 41, 42]. Attentional biases and facilitated threat detection have often been observed in anxiety [5, 10], and are thought to occur at an early stage of information processing and operate at an automatic level [14]. Kenwood et al. [77] have argued that the thalamus, via its connections to the amygdala and prefrontal cortex, may be a contributor to biased threat processing in anxiety, by favoring the transmission of threat signals and filtering out other relevant information. Thus, one can speculate that in high implicit anxiety increased responsivity in the thalamus may be a neural basis of automatic attentional preferences.

By modulating the amygdala, which is connected to the hypothalamus and brainstem [18], the thalamus may also exert an indirect influence on psychophysiological responding to danger signals.

There is evidence that briefly presented and masked emotional faces can evoke spontaneous and involuntary muscle movements in the face of the observer, reflecting automatic mimicry of the seen expression [78, 79]. In our study, masked fearful faces elicited activation in the precentral, middle and superior frontal gyrus. Given their role in action preparation and regulation, and in mirroring observed facial expressions and actions [80–82], activity in the premotor and prefrontal cortex may reflect increased tendency to imitate the non-consciously perceived fearful faces. According to our results, activity in the premotor and lateral prefrontal cortex appears to be particularly increased in individuals with high implicit anxiety. This may imply a more pronounced tendency to mimic the observed expression in anxious individuals. However, we did not use additional electromyography to detect facial muscle movement in our study and this explanation remains speculative. The dorsolateral prefrontal cortex (including also BA8 and BA9) and the precentral gyrus have also been implicated in cognitive control, attentional deployment, and the regulation of emotions (e.g., [77, 83, 84]), for instance via the indirect regulation of amygdalar activity [85]. One can speculate that heightened brain activity in these areas, particularly in highly anxious individuals, may also represent increased regulatory or control efforts during the presence of task-irrelevant threat stimuli. In line with this assumption, Fu et al. [86] and Telzer et al. [87] reported increased lateral prefrontal activity in behaviorally inhibited and anxious children during an attentional control task with threat distractors. The authors have suggested that the heightened activity in control areas may constitute a compensatory mechanism in order to facilitate goal-directed behavior.

We observed in high implicit anxiety increased threat-related processing in subcortical areas that might have provoked a stronger need for cognitive control to maintain the selection of task-relevant information. It is conceivable that automatic control efforts in our study may have targeted the amygdala (see also [86]), where we did not find activation to be increased in response to threat faces or to be modulated by implicit or explicit anxiety. This is in line with earlier studies [62, 88] and supports the notion that successful masking of briefly presented threat faces eliminates amygdalar activation (e.g., [12, 13, 89]). However, based on previous research [12, 13], we expected that the STAI and IAT can independently predict amygdalar responsiveness to subliminal threat, but our hypothesis could not be confirmed. Günther et al. [13] reported moderate effect sizes. It is possible that a relatively small sample size in the present study may explain the lack of significant associations between explicit anxiety and responsivity in the amygdala. Nonetheless, our finding of a processing enhancement in subcortical and cortical brain areas in individuals with a more anxious implicit self-concept extend results from Günther et al. [13] of a relation among implicit anxiety, as defined by misattributions of anxious characteristics in the IPANAT, and automatic responsiveness in the temporal gyrus and amygdala.

Trait anxiety has been described as a vulnerability factor for the development of stress-induced psychopathologies, such as clinical anxiety and depression [6–9]. Thus, Sandi and Richter-Levin [9] have recommended the implementation of prevention programs for highly anxious individuals. The authors have also highlighted the importance of future research to optimize the identification of individuals at risk to develop stress-induced psychopathologies. The anxiety IAT has been shown to be predictive of the onset of anxiety disorders [90] and an unfavorable naturalistic course of pathological anxiety [91]. Therapeutic approaches for the prevention and treatment of anxiety and depression could benefit from the classification of vulnerable individuals based on direct and indirect anxiety measures.

Interestingly, anxious self-associations have been suggested as potential targets in therapeutic intervention programs [91]. Riebel et al. [92] have used evaluative conditioning to alter a negatively biased implicit self-concept in individuals with somatoform complaints. This therapeutic approach may be applied in individuals with high implicit anxiety to foster stronger associations of the self-concept with attributes related to relaxation and calmness. Future studies may investigate the effects of therapeutically modified self-associations on automatic neural threat responses in the fear circuit.

Some limitations of the present study should be noted. Only young and well educated adults were investigated and the results may not be generalizable to older populations with different education levels. Our final sample size of *N* = 37 did not allow us to detect effects with a small or moderate impact. Future studies are needed to replicate our findings and meta-analyses are required to confirm the validity of the results. Until then, our findings should be considered as preliminary. Future studies might implement parametric and non-parametric statistical procedures and cluster correction methods, since the former appear to exaggerate Type I error rates [93], whereas the latter appear to exaggerate Type II error rates [94].

## Conclusions

In sum, our results suggest that individuals respond in the thalamus, precentral gyrus, and lateral prefrontal cortex to non-consciously perceived danger signals dependent on their anxious associative self-representations. From our findings it may be concluded that individuals with an anxious implicit self-concept have a more sensitive threat detection system, which might underlie individual differences in the vulnerability to anxiety disorders. Our data provide evidence that the implicit self-concept of anxiety can significantly predict automatic brain reactivity to fearful faces, and that non-conscious neural processes may underlie biased information processing in anxiety (see also [12]). Given their relative independence, the IAT may be a useful instrument in addition to self-reports to improve the identification of individuals at high risk for the development of psychopathologies or an unfavorable illness course.

## Supplementary Information

**Additional file 1: Figure S1.** IAT design of the five consecutive practice and critical blocks. **Table S1.** Main effects: Brain regions showing activation in response to masked threat faces (at a less stringent cluster-level threshold of puncorrected < 0.05). **Table S2.** Whole-brain regression analyses: Brain regions showing (marginally) significant activation in response to masked threat faces as a function of the implicit self-concept of anxiety (at a less stringent cluster-level threshold of p_uncorrected_ < 0.05). **Table S3.** Non-significant results of regression analyses: Brain regions showing activation in response to masked threat faces as a function of the explicit and implicit self-concept of anxiety (at a very lenient voxel-level threshold of p_uncorrected_ < 0.05 and k>10 voxels, which was considered as non-significant).

## Data Availability

The datasets used and/or analyzed during the current study are available from the corresponding author on reasonable request.
