# Supplementary material for: Non-conscious processing of fear faces: a function of the implicit self-concept of anxiety
Source: BMC Neurosci. 2023 Feb 5;24:12. doi: 10.1186/s12868-023-00781-9 (PMC9901098; doi:10.1186/s12868-023-00781-9)
Supplement: Supplementary file 1 — Additional file 1: Figure S1. IAT design of the five consecutive practice and critical blocks. Table S1. Main effects: Brain regions showing activation in response to masked threat faces (at a less stringent cluster-level threshold of puncorrected < 0.05). Table S2. Whole-brain regression analyses: Brain regions showing (marginally) significant activation in response to masked threat faces as a function of the implicit self-concept of anxiety (at a less stringent cluster-level threshold of puncorrected < 0.05). Table S3. Non-significant results of regression analyses: Brain regions showing activation in response to masked threat faces as a function of the explicit and implicit self-concept of anxiety (at a very lenient voxel-level threshold of puncorrected < 0.05 and k>10 voxels, which was considered as non-significant). [file 12868_2023_781_MOESM1_ESM.pdf]

Additional file 1

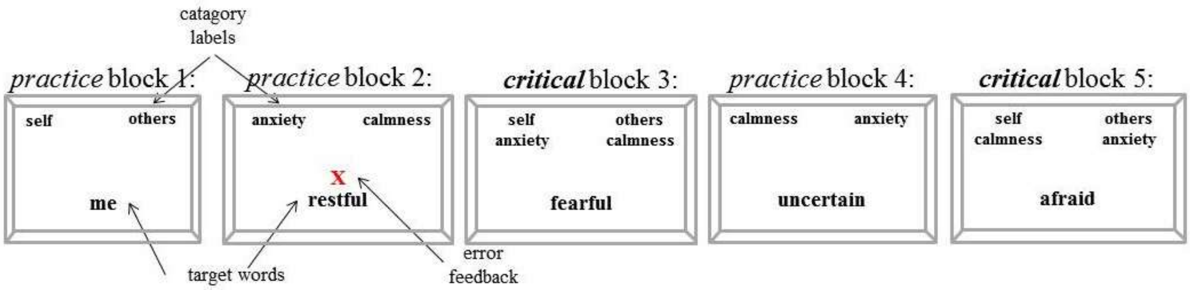

Figure S1: IAT design of the five consecutive practice and critical blocks.

1. Thresholds for the objective visibility check for masked threat faces

Since four emotional conditions (disgust, fear, happy, and neutral) were presented as prime faces, the probability of guessing the correct emotion is 25 %. The experiment included 36 trials for each emotion condition. Thus, a hit rate of 9 trials (with correct emotion responses) would indicate perfect chance performance. But also higher hit rates can be considered as chance performance. According to the binomial distribution (with a 0.25 probability for a success for each trial), which represents our random distribution, a hit rate of 12 trials has a cumulative probability for $P(X>12)$ of 0.09. A hit rate of 13 trials has a cumulative probability for $P(X>13)$ of 0.04. Thus, with a probability of error (p) < 0.05 (one-tailed), 13 hits would significantly differ from the random distribution (i.e., chance performance). Therefore, participants with a hit rate higher than 12 (33.3 %) were considered as objectively aware of the respective emotion, since their hit rate was significantly higher than a mere guessing performance.

2. Parametric and non-parametric correlation analyses for significant clusters

The parametric correlation coefficients (Pearson *r*) for the thalamus and for the frontal gyrus were slightly larger, but not substantially different from the respective non-parametric correlation coefficients (Spearman r_s): $r(35) = 0.57, p < 0.001$ vs. $r_s(35) = 0.53, p < 0.001$ for the cluster in the thalamus and $r(35) = 0.55, p < 0.001$ vs. $r_s(35) = 0.42, p = 0.009$ for the cluster in the precentral gyrus). The overlap between the confidence intervals of all coefficients ($r(35) = 0.57$, 95% CI [0.30, 0.75], $r_s(35) = 0.53$, 95% CI [0.25, 0.73], $r(35) = 0.55$, 95% CI [0.28, 0.74] and $r_s(35) = 0.42$, 95% CI [0.11, 0.65]) suggests that the correlations do not significantly differ from each other.

Table S1 Main effects: Brain regions showing activation in response to masked threat faces (at a less stringent cluster-level threshold of $p_{uncorrected} < .05$)

	Hemisphere	Peak *T*-value	Peak-level $p_{uncorrected}$	Cluster size (voxels)	Cluster-level $p_{uncorrected}$	Peak MNI		
						x	*y*	*z*
Disgust > neutral								
Inferior frontal extending to precentral gyrus	L	3.99	<.001	108	.03	-45	14	25
Cuneus (BA 18/19)	L	3.96	<.001	150	.01	-3	-82	28
Lingual extending to posterior cingulate gyrus	R	3.69	<.001	124	.02	15	-58	-2
Middle temporal gyrus (BA22) extending to supramarginal gyrus (BA40)	L	3.60	<.001	90	.04	-39	-61	13
Fear > neutral								
Supramarginal gyrus extending to precentral gryrus (BA6), suppl. motor area, postcentral gyrus, superior and middle frontal gyrus	L	4.97	<.001	858	<.001	-30	2	64
Thalamus extending to parahippocampal gyrus, middle temporal gyrus, posterior cingulate gyrus	L/R	4.58	<.001	896	<.001	9	-31	7

Table S2 Whole-brain regression analyses: Brain regions showing (marginally) significant activation in response to masked threat faces as a function of the implicit self-concept of anxiety (at a less stringent cluster-level threshold of $p_{uncorrected}$ < .05)

	Hemi-sphere	Peak T-value	Peak-level $p_{uncorrected}$	Cluster size (voxels)	Cluster-level $p_{uncorrected}$	Peak MNI		
						x	y	z
Disgust vs. neutral								
Precuneus (BA7) extending to cingulate gyrus	L/R	3.31	.001	73	.06	-3	-40	34
Fear vs. neutral								
Supramarginal gyrus	L	4.97	<.001 (p_{FWE} = .07)	71	.07	-48	-55	31
Thalamus	L/R	4.66	<.001	263	.002	-6	-13	-1
Precentral gyrus, extending to middle and superior frontal gyrus (BA6,8,9)	L	4.18	<.001	299	.001	-42	8	40
Cingulate gyrus (BA31) extending to precuneus, and cuneus	L/R	3.70	<.001	143	.02	-6	-61	28
Middle and inferior frontal gyrus (BA9)	R	3.12	<.001	80	.06	36	17	31
Superior temporal gyrus extending to insula	R	3.14	.002	89	<.05	51	-28	10

Table S3 Non-significant results of regression analyses: Brain regions showing activation in response to masked threat faces as a function of the explicit and implicit self-concept of anxiety (at a very lenient voxel-level threshold of $p_{uncorrected}$ < .05 and k>10 voxels, which was considered as non-significant)

	Hemi-sphere	Peak *T*-value	Peak-level $p_{uncorrected}$	Cluster size (voxels)	Peak MNI		
					x	*y*	*z*
**Explicit anxiety (STAI)**							
Disgust vs. neutral							
Positive correlation							
Middle frontal gyrus	R	2.98	.003	60	27	38	-2
Middle frontal gyrus	L	2.97	.004	18	-36	11	58
Superior frontal gyrus	L	2.71	.005	37	-12	20	43
Temporal lobe sub-gyral extending to superior temporal gyrus	L	2.62	.006	40	-33	-58	19
Parietal lobe sub-gyral	R	2.57	.007	35	24	-52	22
Superior medial frontal	L/R	2.53	.008	95	0	44	28
Temporal lobe sub-gyral	L	2.08	.02	13	-42	-52	1
Negative correlation							
Right middle occipital gyrus and calcarine (BA18) extending to left cuneus (BA17)	R/L	3.73	<.001	194	27	-76	7

(Table S3 continuation)	Hemi-sphere	Peak *T*-value	Peak-level $p_{uncorrected}$	Cluster size (voxels)	Peak MNI *x*	*y*	*z*
Negative correlation (continuation)							
Supramarginal gyrus extending to inferior parietal	L	3.10	.002	54	-51	-49	34
Parietal lobe, sub-gyral, inferior parietal	R	2.64	.006	29	30	-40	31
Frontal lobe, sub-gyral	R	2.24	.02	30	27	14	19
Middle cingulate gyrus	L	2.17	.02	18	-6	-31	37
Cingulate gyrus	L	2.06	.02	11	-12	-40	28
Frontal lobe, sub-gyral	R	2.06	.02	16	27	-22	40
Fearful vs. neutral							
Positive correlation							
Superior frontal gyrus (BA8)	L	2.99	.003	291	-12	23	43
Precentral gyrus	R	2.80	.004	135	39	-16	61
Middle frontal gyrus (BA10)	R	2.76	.005	111	39	47	1
Temporal sub-gyral extending to middle occipital gyrus	L	2.71	.005	73	-39	-52	1
Precuneus	R	2.60	.007	40	21	-52	22

(Table S3 continuation)	Hemi-sphere	Peak *T*-value	Peak-level $p_{uncorrected}$	Cluster size (voxels)	Peak MNI		
					x	*y*	*z*
Positive correlation (continuation)							
Fusiform gyrus	L	2.49	.009	18	-18	-4	43
Postcentral gyrus	R	2.48	.009	24	57	-16	40
Calcarine sulcus	R	2.46	.009	15	30	-61	4
Insula	L	2.44	.01	11	-33	26	4
Cingulate Gyrus	R	2.43	.01	12	15	-1	46
Cuneus	L	2.22	.02	12	-9	-64	25
Temporale lobe, sub-gyral	L	2.08	.02	23	-33	-58	25
Parietal lobe	L	2.06	.02	14	-42	-22	25
Negative correlation							
Superior frontal gyrus	L	2.23	.01	18	-15	44	22

(Table S3 continuation)	Hemi-sphere	Peak *T*-value	Peak-level $p_{uncorrected}$	Cluster size (voxels)	Peak MNI *x*	*y*	*z*
**Implicit anxiety (IAT)**							
Disgust vs. neutral							
Positive Correlations							
Superior frontal gyrus	R	4.38	<.001	87	18	35	52
Right lingual gyrus, extending to left and right middle cingulate gyrus, left superior temporal gyrus	R/L	3.45	.001	1361	12	-43	-2
Middle frontal gyrus (BA9)	L	3.35	.001	241	-45	11	37
Superior temporal and angular gyrus, extending to inferior parietal (BA40) and middle temporal gyrus (BA22)	L	3.26	.001	97	-48	-61	25
Angular gyrus	R	2.88	.003	77	45	-46	28
Inferior frontal extending to middle frontal gyrus	R	2.81	.004	49	30	14	40
Precentral gyrus	R	2.80	.004	103	27	-25	52
Superior frontal gyrus (BA8)	L	2.54	.008	31	-6	35	58
Postcentral gyrus (BA2)	L	2.48	.009	20	-51	-31	55
Postcentral gyrus	R	2.35	.01	11	57	-7	25

**(Table S3 continuation)**	Hemi-sphere	Peak *T*-value	Peak-level $p_{uncorrected}$	Cluster size (voxels)	Peak MNI *x*	*y*	*z*
Positive correlations (continuation)							
Paracentral gyrus (BA6)	L	2.34	.01	80	-6	-22	73
Postcentral gyrus	L	2.24	.02	61	-51	-13	31
Superior temporal gyrus (BA13)	R	2.21	.02	83	42	-31	13
Middle cingulate gyrus (BA24)	L	2.09	.02	31	-9	-7	46
Negative correlations							
Frontal lobe, sub-gyral extending to anterior cingulate gyrus	R	2.97	.003	64	21	-7	43

Of note, in this table, only regression results for contrasts are reported where no significant associations with explicit or with implicit anxiety were found.
